# Bibliometric Insights Into Thromboelastography Research Using PubMed and VOSviewer

**DOI:** 10.7759/cureus.76160

**Published:** 2024-12-21

**Authors:** Vamsi Krishna Uppalapati, Deb Sanjay Nag, Rishi Anand, Amlan Swain, Seelora Sahu, Himanshu Kumar

**Affiliations:** 1 Department of Anesthesiology, Tata Main Hospital, Jamshedpur, IND; 2 Department of Anesthesiology, Manipal Tata Medical College, Jamshedpur, IND

**Keywords:** bibliometric analysis, coagulopathies, personalized medicine, pubmed database, thromboelastography (teg), vosviewer

## Abstract

Thromboelastography (TEG) has evolved from a primarily surgical tool to a key instrument in broader medical fields, including personalized medicine for coagulopathies. The rationale for conducting this bibliometric analysis of TEG is to understand the evolution and current state of research in this critical field. By identifying publication trends, key contributors, and major developments, this study aims to provide valuable insights to guide future research and clinical practices in TEG. This bibliometric study, utilizing the PubMed database, delineates the research landscape of TEG by analyzing publication patterns, the frequency of keywords, and author collaborations to understand the growth and direction of the discipline. Bibliographic data were extracted from PubMed, encompassing publications up to the year 2024. Using VOSviewer software, network maps were generated to visualize relationships among authors, institutions, and thematic keywords. Rigorous selection criteria were applied, focusing on peer-reviewed articles relevant to TEG's medical and surgical applications, while excluding non-English papers, non-peer-reviewed materials, and non-clinical research. The analysis identified a significant increase in TEG-related publications in recent years: 461 in 2021, 427 in 2022, and 315 in 2023, with 6 publications early in 2024. Key figures such as Moore (111 publications), Nielsen (77 publications), and Moore (71 publications) emerged as prolific contributors. The research also highlighted a heightened focus on demographic-specific characteristics in TEG studies, reflecting the shift toward personalized medicine. This bibliometric analysis provides a comprehensive overview of TEG research, signifying its growing clinical relevance and potential for future applications. Despite limitations, such as potential database selection bias, the study outlines a clear expansion of TEG into various clinical environments and underscores the importance of inter-author collaboration. It suggests that TEG research is progressing towards interdisciplinary applications, including genetic profiling and machine learning, to further enhance patient-specific treatment modalities.

## Introduction and background

Thromboelastography (TEG) is a complex laboratory test that evaluates blood clotting characteristics, identifies abnormalities in blood clotting, and helps guide decisions on the use of blood products. TEG offers a comprehensive assessment of a patient's coagulation status by quantifying the viscoelastic properties of blood during clot formation. This feature is especially advantageous for detecting coagulopathy and optimizing the administration of blood products in different groups of patients. TEG, traditionally used mainly in surgical patient care, has been extended to broader medical contexts. The use of this technology has had a significant effect on the treatment of severely injured trauma patients and those with liver disease [1-3]. It has played a crucial role in improving the efficient use of blood products and increasing the chances of survival. During the COVID-19 pandemic, TEG has been essential in monitoring coagulation issues. This has resulted in the creation of customized TEG-guided anticoagulation strategies for critically ill individuals [4].

TEG has demonstrated its worth in pediatric neurosurgery by effectively identifying coagulation problems that conventional laboratory diagnostics may overlook. Moreover, within the urosepsis and non-trauma critical care field, TEG has played a crucial role in enhancing our comprehension of the clinical importance of its parameters and has guided the development of more precise transfusion techniques [5]. Specifically, in critical care situations unrelated to trauma, the use of TEG-guided transfusion techniques has resulted in higher utilization of blood products when compared to standard coagulation tests. The adaptability of TEG in several medical fields is becoming more visible as it continues to evolve, contributing to improved patient care and clinical results [6]. Bibliometric study is vital in understanding research trends and how knowledge is shared in TEG and other medical instruments and application areas. It includes the number of analyzed written works, allowing researchers to identify new research subjects, potential collaborators, and key publishing venues. By using different computer programs like CiteSpace, VOSviewer, and R-Bibliometric, scientists can create visualizations such as link maps and time charts that show data about countries, institutions, authors, and journals. These images help in understanding the body of knowledge based on commonly used terms related to a specific topic [7,8]. This provides valuable insights into the growth of TEG, aiding researchers and decision-makers in understanding current developments and future directions.

Bibliometric analysis serves as a crucial tool in mapping the research landscape of TEG. It helps identify publication trends, key contributors, and emerging research themes. Understanding these aspects is vital for guiding future research directions, fostering collaborations, and enhancing clinical practices in TEG. This bibliometric study seeks to analyze publication patterns, identify influential studies, and reveal new trends in TEG literature. By conducting a thorough analysis of data obtained from PubMed and visualized using VOSviewer, this study aims to create a comprehensive overview of TEG research, identify the main contributors, and predict future trends in the area.

## Review

Methodology

Data Source

This bibliometric study uses PubMed as the primary data source to extract bibliographic information. PubMed is well known for its vast life science and biomedical literature collection, offering various articles, reviews, and other academic papers. The study utilizes PubMed's extensive search capabilities and database to retrieve numerous relevant publications on TEG. This facilitates a meticulous aggregation and examination of data, ensuring a comprehensive and inclusive selection of the available literature, which is essential for a precise and all-encompassing bibliometric analysis. The search used the principal term "Thromboelastography," ensuring a focused and precise approach to retrieving publications on the subject. The search may have been further refined by incorporating additional keywords and Boolean operators to focus on certain aspects of TEG, such as its medical or surgical applications.

Time frame: The literature search encompassed a broad spectrum of publication years, as indicated by the dataset, which includes publications up to 2024. This extensive period guarantees the incorporation of fundamental studies and the most recent advancements in research.

Filters employed: Although the specific filters utilized during the search are not explicitly mentioned in the given data, standard filters could include publication type (e.g., journal articles, reviews), language (e.g., English), and article status (e.g., peer-reviewed). The goal is to prioritize high-quality, relevant, and readily available literature.

Data extraction: The records obtained from PubMed were gathered and organized into a dataset that includes important bibliographic details such as PMID (PubMed identifier), title, authors, citation information, publication year, and DOI. This dataset forms the foundation for future bibliometric studies conducted with VOSviewer.

Temporal analysis: A supplementary dataset presents an annual tally of publications on TEG, demonstrating increasing interest and research productivity over time, particularly in recent years. Examining this trend is essential for understanding the development of the field and identifying periods of notable research activity.

The search strategy's rigorous design ensures an intensive and all-encompassing dataset, providing a solid foundation for the thorough bibliometric analysis of TEG literature using VOSviewer. The study aims to uncover publication patterns, influential research, and emerging trends, offering valuable insights into the landscape and future direction of the field.

Inclusion and Exclusion Criteria

The primary inclusion criteria for the bibliometric analysis focused on peer-reviewed articles and reviews explicitly addressing TEG. The selection of publications focused on their relevance to TEG's medical and surgical applications, focusing on studies that offered substantial insights into its methodology, outcomes, and breakthroughs. The exclusion criteria disregarded non-English papers, non-peer-reviewed material such as commentaries and editorials, and research not directly relevant to TEG's clinical or theoretical elements. The rigorous selection method guarantees a concentrated and top-notch dataset, enabling a thorough and enlightening analysis of the TEG literature landscape.

While PubMed was chosen for its extensive coverage of biomedical literature, the exclusive reliance on this database may introduce selection bias, potentially excluding significant research indexed in other databases such as Scopus or Web of Science. Additionally, the exclusion of non-English publications may lead to language bias, omitting valuable contributions from non-English-speaking regions. Furthermore, the exclusion of gray literature, such as conference papers and technical reports, could limit the comprehensiveness of the analysis.

Bibliometric Tools and Software

VOSviewer is a powerful bibliometric software program for building and displaying bibliometric networks. These networks consist of journals, researchers, or individual articles, represented visually using metrics such as co-citation, bibliographic coupling, or co-authorship. VOSviewer is proficient at uncovering patterns and connections by creating intricate network maps. These maps effectively emphasize the interactions among different entities within a research domain. It is highly efficient for examining co-authorship networks to comprehend collaboration patterns, co-citation networks to recognize prominent works, and keyword occurrences to spot emerging trends and thematic structures within the scientific literature.

Analytical Framework

The VOSviewer analytical framework was utilized to extract pertinent bibliometric information, such as citations, keywords, and authorship details, from the PubMed dataset. The processed data was subsequently loaded into VOSviewer and underwent normalization to ensure comparability. The software generated network diagrams by analyzing co-authorship, co-citation, and keyword co-occurrence, which provided insights into the structure and evolution of the TEG research domain. Clusters were detected, signifying cohesive groups of articles or writers. The visualization depicted the robustness of connections and the significance of items in the network, enabling a clear and thorough comprehension of the study landscape.

Results

Overview of Literature

The bibliometric analysis in the "Overview of Literature" presented in Table 1 unveiled some fundamental attributes of TEG studies. This table indicates a substantial body of research in TEG, highlighting the number of publications per year. The temporal distribution of publications demonstrates a sustained research focus on TEG, with a significant surge in recent times. In particular, 461 papers were published in 2021, 427 in 2022, and 315 in 2023. The number decreased to 6 in the early months of 2024, indicating a dynamic and expanding area of research. Some authors distinguish themselves in this field for their abundant and noteworthy contributions, presented in Table 2. Moore is the leading author with 111 publications, followed by Nielsen with 77 and Moore with 71. Additional noteworthy contributors include Johansson, Banerjee, and Wade, all of whom have significantly contributed to the existing body of literature. These data emphasize TEG research's active and collaborative characteristics, where a small number of individuals and their affiliated institutions play a significant role in the scholarly discussion.

**Table 1:** Publication Count Over the Past 5 Years in Thromboelastography

Year	Publication Count
2024	6
2023	315
2022	427
2021	461

**Table 2:** Top 10 Prolific Authors in Thromboelastography

Author	Publication Count
Moore EE	111
Nielsen VG	77
Moore HB	71
Johansson PI	59
Banerjee A	50
Wade CE	50
Nielsen VG	46
Holcomb JB	43
Sauaia A	39
Silliman CC	38

Table 3 presents the journals with the highest number of articles on TEG, indicating "Blood Coagul Fibrinolysis" as the leading publication venue with 227 articles. It is followed by "Anesth Analg" and "Thromb Res" with 199 and 186 articles, respectively, showcasing these journals as central platforms for disseminating research in this field.

**Table 3:** Top 10 Most Featured Journals in Thromboelastography

Journal/Book	Article Count
Blood Coagul Fibrinolysis	227
Anesth Analg	199
Thromb Res	186
J Trauma Acute Care Surg	158
Transfusion	129
J Cardiothorac Vasc Anesth	125
Thromb Haemost	119
Br J Anaesth	108
Semin Thromb Hemost	97
J Thromb Haemost	95

These tables provide a concise view of the publication trends, influential authors, and prominent journals within TEG research. They highlight the significant contributors and platforms that drive the scholarly discourse in this area.

Co-authorship Analysis

**Figure 1:**
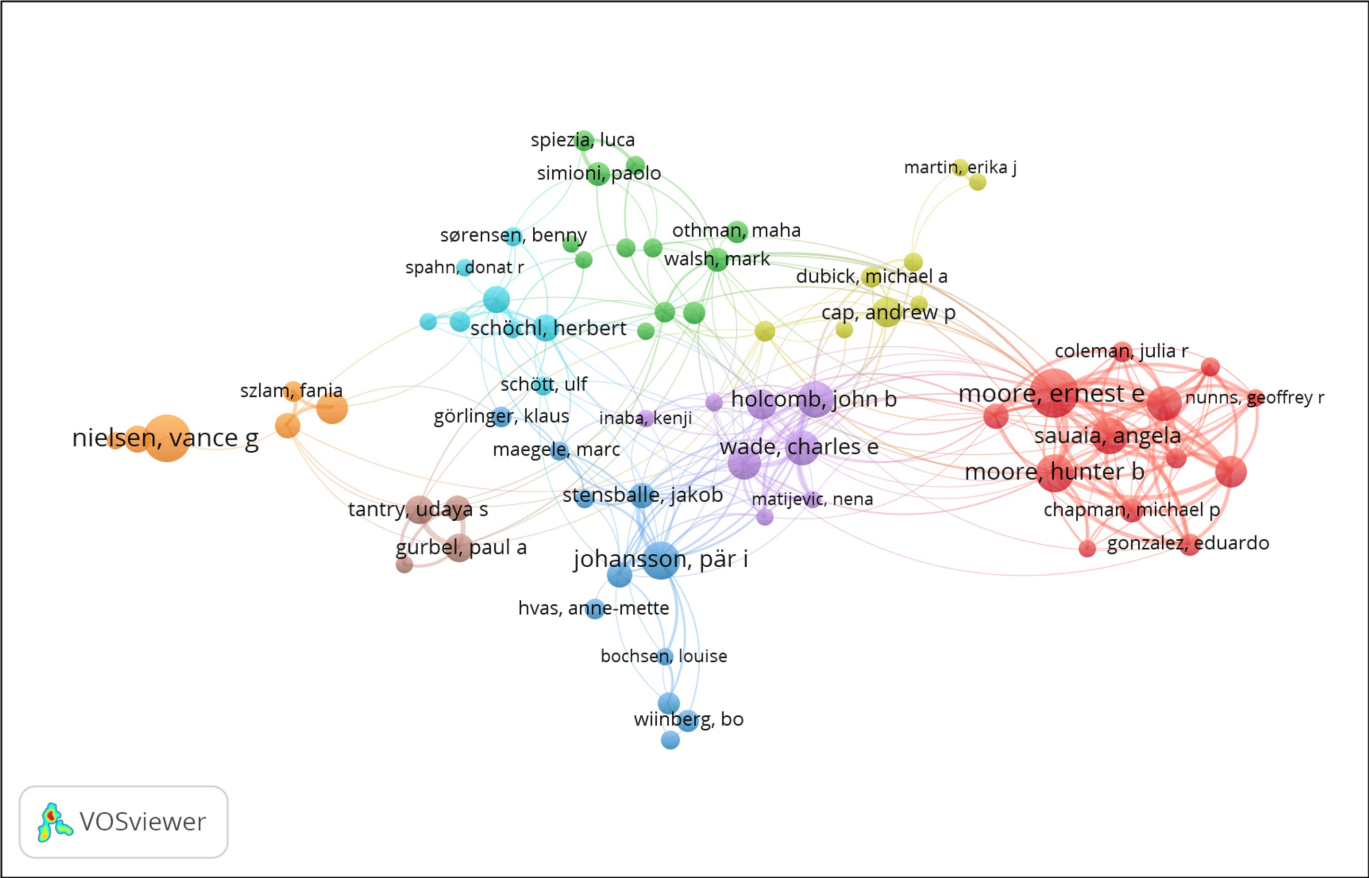
Co-Authorship Network in Thromboelastography Research

The VOSviewer network visualization in Figure 1 depicts a co-authorship analysis conducted in the study domain. The nodes represent individual authors, with the size of each node indicating the number of publications or the extent of their contribution to the area. Connections between nodes represent co-authorship, with thicker lines indicating more frequent collaboration. The different hues represent separate clusters of authors who tend to collaborate. The compact orange cluster in the lower-left quadrant most likely indicates a cohesive community of scholars characterized by extensive collaboration, potentially reflecting a specialized study area or a highly productive research team.

Keyword and Term Analysis

**Figure 2:**
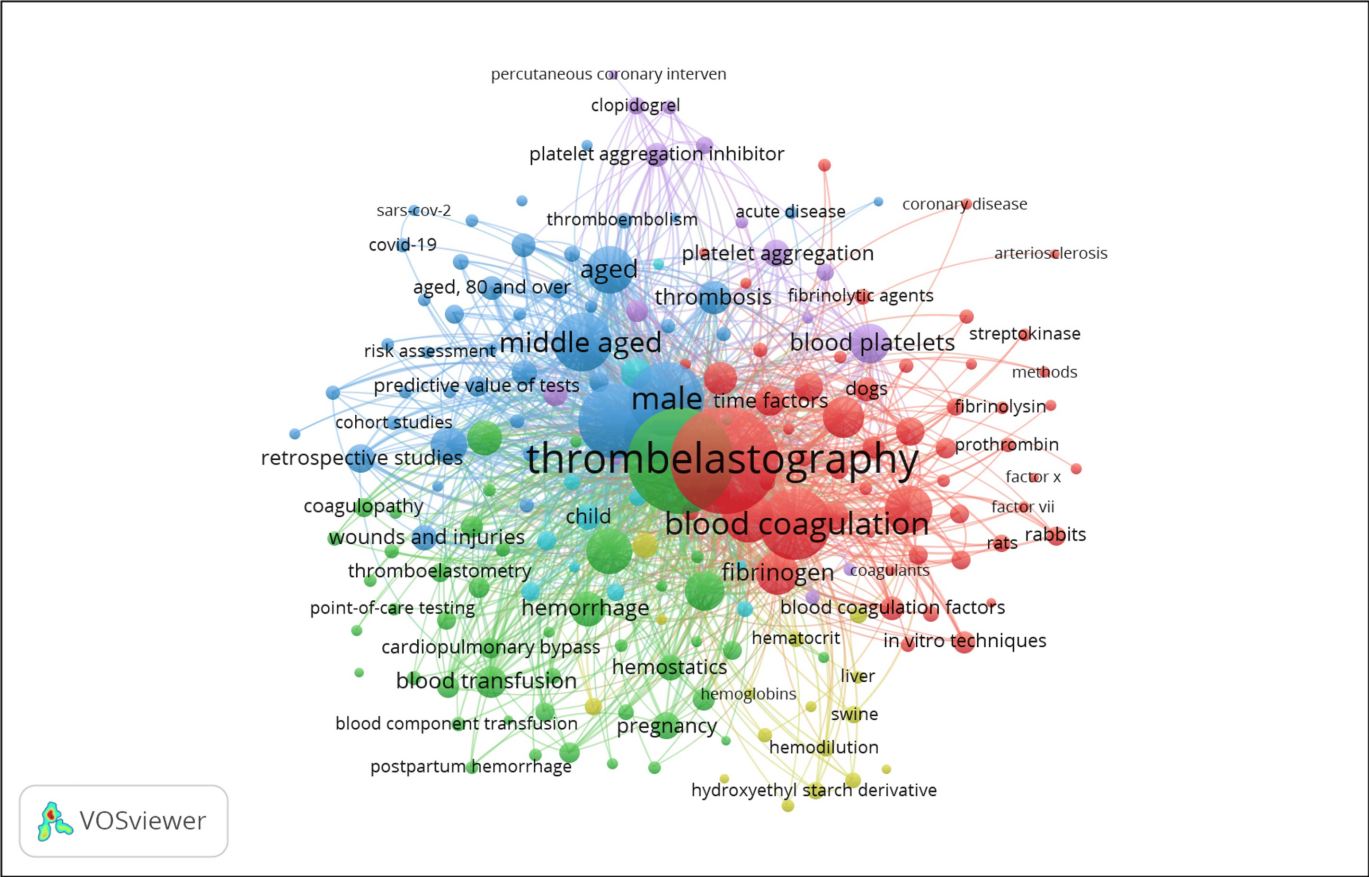
Keyword Co-occurrence Network in Thromboelastography Research

The network visualization in Figure 2 depicts the co-occurrence of keywords in TEG research, as analyzed using VOSviewer. The different colors reflect separate thematic clusters, and the size of each node indicates the frequency of each keyword's occurrence in the literature. The presence of central, prominent terms such as "thromboelastography," "blood coagulation," and "fibrinogen" indicates that these are the primary areas of research interest. The close association between nodes such as "male" and "aged" with "thromboelastography" suggests that demographic characteristics are frequently considered in research. The lines linking the nodes represent the keywords that often appear together. Thicker lines indicate stronger correlations, reflecting closely connected research topics.

The bibliometric analysis performed using VOSviewer has provided a comprehensive overview of TEG research, emphasizing essential thematic clusters and highly productive contributors. The presence of particular terms close to central study issues highlights the focused yet dynamic nature of the field.

Discussion

The utilization of VOSviewer software in bibliometric analysis provides a detailed and complex perspective on the study domain of TEG, delivering critical insights into the thematic and collaborative structure of the field. The results indicate a strong emphasis on essential subjects such as "blood coagulation," "fibrinogen," and demographic variables like "age" and "gender," which are crucial to the investigation of hemostasis and thrombosis. This highlights the clinical significance of TEG in customizing care methods specific to each patient, especially in populations with diverse coagulation profiles.

Examining trends and patterns indicates increased publications and research activity, reflecting a growing interest in TEG. The temporal publishing trend shows a significant increase in research output in recent years, which may be attributed to technological developments in TEG devices and a broader comprehension of its applications beyond surgery, extending into obstetrics and intensive care areas. Furthermore, the detected keywords indicate a transition from fundamental scientific research to more tangible, applied uses of TEG, suggesting that the field is advancing and becoming more focused on providing direct medical assistance to patients.

The bibliometric study identifies the TEG discipline's most influential researchers and writers. Notable individuals such as Moore, Nielsen, and Moore play crucial roles in advancing TEG research through their significant contributions and collaborations. These highly productive authors often operate as central points connecting different specialized areas and promoting the incorporation of TEG into wider therapeutic use. Furthermore, significant journals like "Blood Coagulation and Fibrinolysis" and "Anaesthesia and Analgesia" play an essential role in disseminating cutting-edge TEG research.

This study has certain drawbacks. Despite its extensive coverage, the exclusive dependence on a solitary database could lead to the exclusion of pertinent papers cataloged in other sources, potentially basing the study on specific demographics or research areas. In addition, the analysis is constrained by the selection of keywords and indexing terms included in the database, potentially failing to encompass the entirety of TEG research, particularly novel terminology or concepts. The search strategy for this study focused exclusively on TEG and did not include related terms and technologies such as rotational thromboelastometry (ROTEM). This omission may have limited the scope of the analysis, potentially overlooking comparative studies and innovations involving other coagulation testing technologies. Including related terms and technologies in future analyses could provide a more comprehensive overview of the field and highlight collaborative research efforts and advancements.

Based on these data, numerous directions for TEG research can be inferred, indicating future trends. The growing emphasis on demographic characteristics in TEG studies suggests the rise of personalized medicine as a rapidly developing study area. Future studies may be motivated by the necessity to create customized coagulation management strategies for various patient populations, including pediatrics and geriatrics. Furthermore, the utilization of TEG in non-surgical settings, such as the surveillance of coagulation abnormalities in systemic illnesses or during extended periods of anticoagulant treatment, has the potential to broaden. With the emergence of precision medicine, TEG could be combined with genetic profiling to enhance the customization of treatment strategies for individual patients.

The network visualizations indicate a rise in collaboration between different fields, implying the possibility of interdisciplinary research endeavors that could result in creative uses of TEG. Integrating TEG data with machine learning can enhance predictive models for bleeding risks or transfusion needs in complex clinical situations. To summarize, the bibliometric results of this study offer a valuable structure for comprehending the historical and current state of TEG research while also setting the stage for future investigations. In the future, the TEG research community can increase its influence on healthcare and patient outcomes by further developing its network of authors, institutions, and journals.

## Conclusions

The utilization of VOSviewer in conducting bibliometric analysis has provided a comprehensive and detailed view of TEG, clearly illustrating the research's history and expanding scope. The data show a substantial increase in papers linked to TEG, demonstrating the growing acknowledgment of its clinical usefulness. The discussion's central focus is blood clotting and individual patient characteristics, highlighting the transition toward personalized medicine. The influential role of prominent writers and journals has been crucial in shaping the research landscape, serving as foundational pillars that facilitate the spread of novel knowledge within the discipline. Although the research provides vital insights, it also recognizes several limitations, such as the possibility of bias in the database and the exclusion of newly emerging concepts that are not yet widely discussed in the literature. However, the observed patterns indicate a promising future for TEG research, where integrating several fields of study could reveal new medical applications and improve patient treatment. As the field advances, sophisticated analytical methods, such as machine learning with TEG data, can potentially enhance predictive models tailored to the unique requirements of various patient populations. This bibliometric analysis provides valuable insights into the research landscape of TEG, highlighting its growing clinical relevance and key contributors. However, the study acknowledges potential limitations such as database selection bias, language bias, and the exclusion of gray literature. Future research should aim to include multiple databases, non-English publications, and gray literature to ensure a more comprehensive understanding of the field. Additionally, incorporating related technologies like ROTEM could further enrich the analysis. The bibliometric analysis not only provides an overview of the current status of research on TEG but also offers guidance for future studies in the field of hemostatic control, highlighting prospective areas that have not yet been explored.
